# A Rare Case of Extranodal Rosai-Dorfman Disease Involving the Entire Colon

**DOI:** 10.7759/cureus.98354

**Published:** 2025-12-03

**Authors:** Pravin R Suryawanshi, Neha D Borde, Sushil S Deshpande

**Affiliations:** 1 Department of Hepato Pancreato Biliary Surgery, MGM (Mahatma Gandhi Mission) Medical College and Hospital, Aurangabad, IND; 2 Department of Pathology, MGM (Mahatma Gandhi Mission) Medical College and Hospital, Aurangabad, IND

**Keywords:** case report, colon involvement, duodenal involvement, extranodal rdd, gastrointestinal histiocytosis, gastrojejunostomy, rare disease, rosai-dorfman disease, total colectomy

## Abstract

Previously known as sinus histiocytosis accompanied by massive lymphadenopathy, Rosai-Dorfman Disease (RDD) is an extremely uncommon idiopathic histiocytic condition that does not have a malignant cause. In young patients, RDD often manifests as a painless cervical lymphadenopathy. However, some cases present as the extra-nodal variant of the disease, which might show a mass in the skin, soft tissue, nasal cavity, eye, bone, or other sites. RDD's involvement in the gastrointestinal (GI) tract is exceedingly uncommon. We reported a case of extra-nodal RDD affecting the GI tract in a 47-year-old Indian male patient who presented with chronic lower abdominal pain and constipation. On computed tomography, there was circumferential thickening of the ascending, transverse, and descending colon and recto-sigmoid junction with luminal narrowing and pericolic enlarged lymph nodes. Similar short segment lesions were noted in the duodenum without luminal narrowing. Colonoscopy showed luminal narrowing at the sigmoid colon with inflammatory changes of the mucosa. After receiving treatment at different centres for almost a year, the patient underwent total abdominal colectomy with ileorectal anastomosis and was diagnosed with RDD, extra-nodal type. Microscopy showed histiocytic disease with transmural involvement of the entire colon and surrounding mesentery. The histiocytes were negative for c-kit but positive for S100 and CD68. Related literature was reviewed and compared with this case. The patient is regularly followed up in the outpatient department and is under remission for four years of follow-up.

## Introduction

A non-malignant proliferation of characteristic histiocytic cells within lymph node sinuses and lymphatics at extranodal locations is a hallmark of Rosai-Dorfman Disease (RDD), a rare condition known as sinus histiocytosis with extensive lymphadenopathy [1]. In 1969, Rosai and Dorfman demonstrated it as an entirely distinct clinicopathologic entity [2]. It occurs worldwide and is primarily a disease of childhood and young adults. 

Among others, axillary, para-aortic, inguinal, and mediastinal lymph nodes are commonly affected. Extranodal disease is documented in less than half of patients, with or without associated lymphadenopathy, which possibly develops later during the course of the disease. Practically, every organ system can be affected, but the most commonly affected extranodal sites include the central nervous system, skin and soft tissues, retina, the upper respiratory tract, and the skeletal system, followed by the genitourinary and lower respiratory tracts and the oral cavity. The gastrointestinal (GI) tract is the least commonly involved site [3]. This report describes a rare presentation of RDD affecting the GI tract.

## Case presentation

A 47-year-old male patient presented with lower abdominal pain and chronic constipation. He was in good health till a year prior to presentation. There was no weight loss, nausea, vomiting, diarrhoea, melena, or jaundice. There was no evidence of any clinical lymphadenopathy. The patient had a known history of hypertension with well-controlled blood pressure on medication.

The patient had been undergoing treatment for the same at various institutes under the assumption of various different diagnoses, including ulcerative colitis, without any relief, and was later diagnosed with infective colitis with a component of melanosis coli and received antibiotics and laxatives, which provided symptomatic relief for a few days, but the symptoms recurred. Multiple colonoscopies were done over the year, which showed the progressive nature of the disease and lymphoplasmacytic and macrophage infiltration on biopsy, but no specific pathology. The patient had multiple episodes of subacute obstruction, which caused severe dehydration and led to acute kidney injury due to acute tubular necrosis, and was under intensive care for the same. With proper hydration and dialysis, the patient completely recovered from acute kidney injury (AKI).

**Figure 1:**
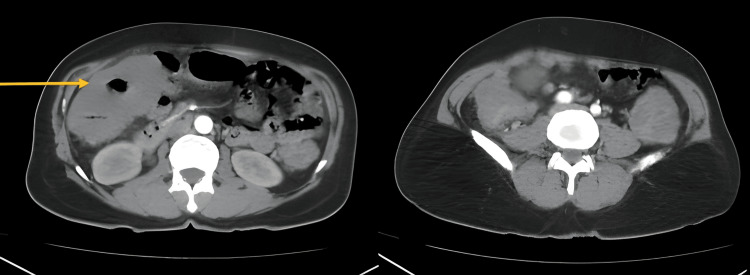
CT image showed short segment circumferential thickening of D1 and D2 was noted.

The patient underwent contrast-enhanced computed tomography at our institute, which showed long-segment polypoidal circumferential thickening in the ascending, transverse, and descending colon, and rectosigmoid junction with skip areas in between, producing luminal narrowing and loss of mural stratification with enlarged pericolic lymph nodes. Similar morphology with short segment circumferential thickening of D1 and D2 was noted (Figure 1).

**Figure 2:**
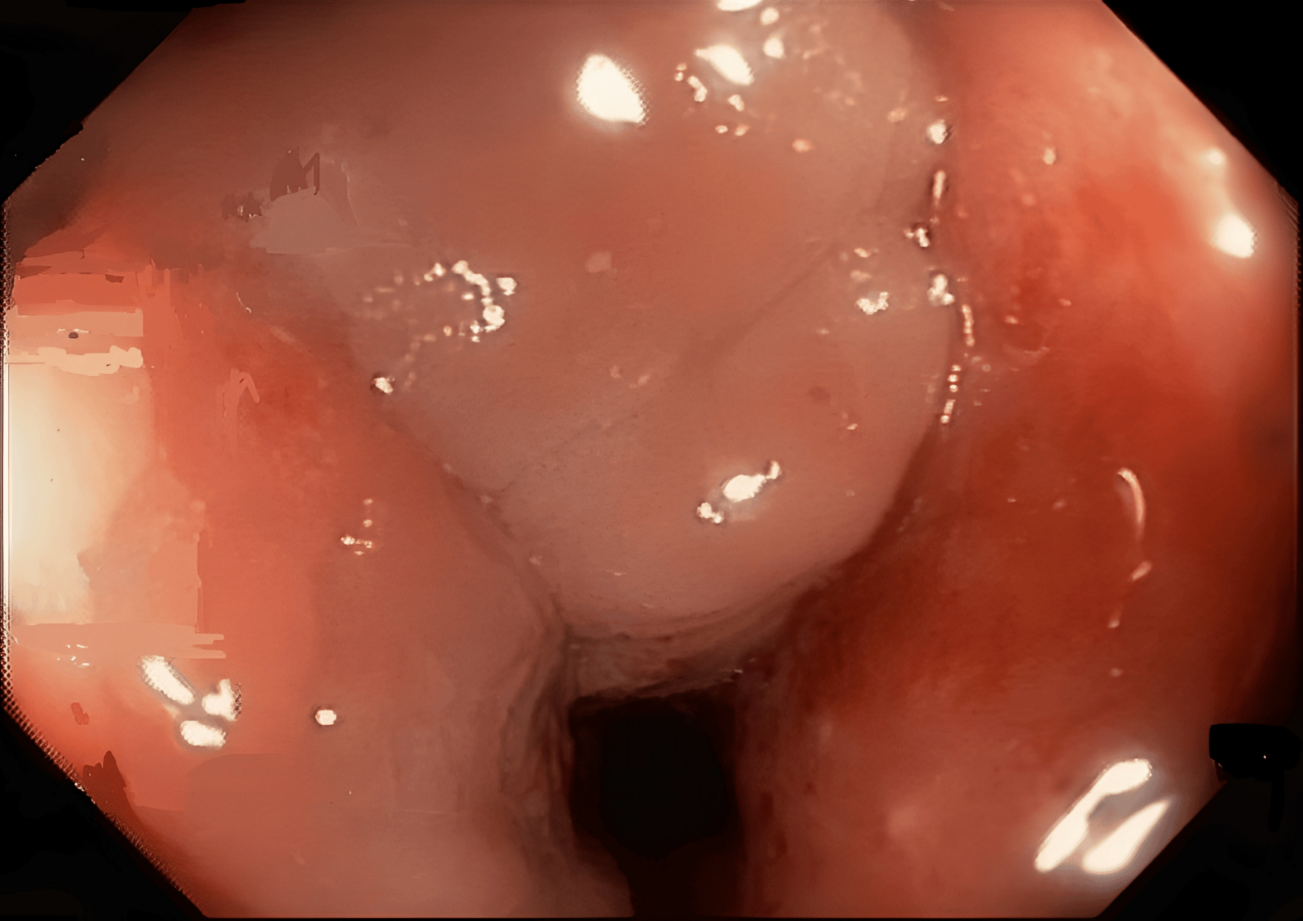
Colonoscopy image showed luminal narrowing of sigmoid colon with inflammatory changes in mucosa with minimal pigmentation beyond which scope could not be negotiated.

Colonoscopy was done, which showed luminal narrowing of the sigmoid colon with inflammatory changes in the mucosa with minimal pigmentation beyond which the scope could not be negotiated (Figure 2).

**Figure 3:**
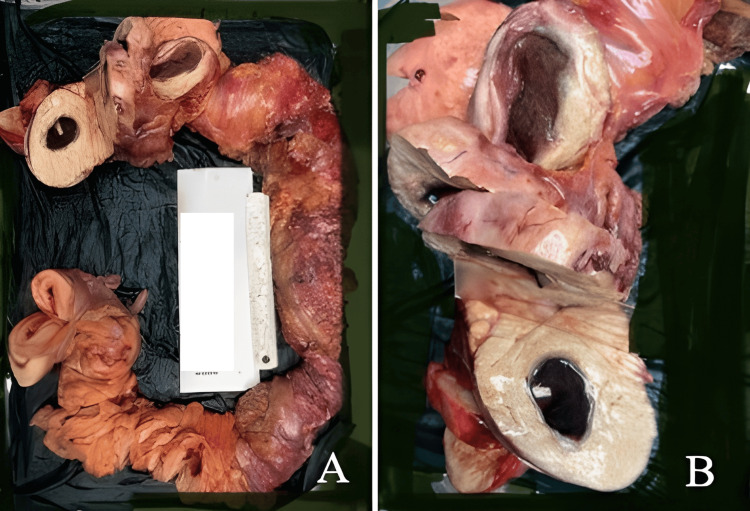
(A) Evidence of bosselation along the length of the entire colon with tumor-like circumferential hypertrophy at the ceacum and proximal ascending colon, hepatic flexure, distal descending colon and recto-sigmoid. (B) Cut surface of colon reveals firm thickened yellowish wall

Since the mucosa was not showing any pathology but had significant thickening, a core needle biopsy was done, which was reported as colonic xanthomatosis. In view of significant luminal narrowing with repeated episodes of subacute obstruction and core needle biopsy of colonic xanthomatosis, the patient was planned for surgical intervention. Intra-operatively, there was evidence of bosselation along the length of the entire colon with tumor-like circumferential hypertrophy at the ceacum and proximal ascending colon, hepatic flexure, distal descending colon, and recto-sigmoid (Figure 3).

**Figure 4:**
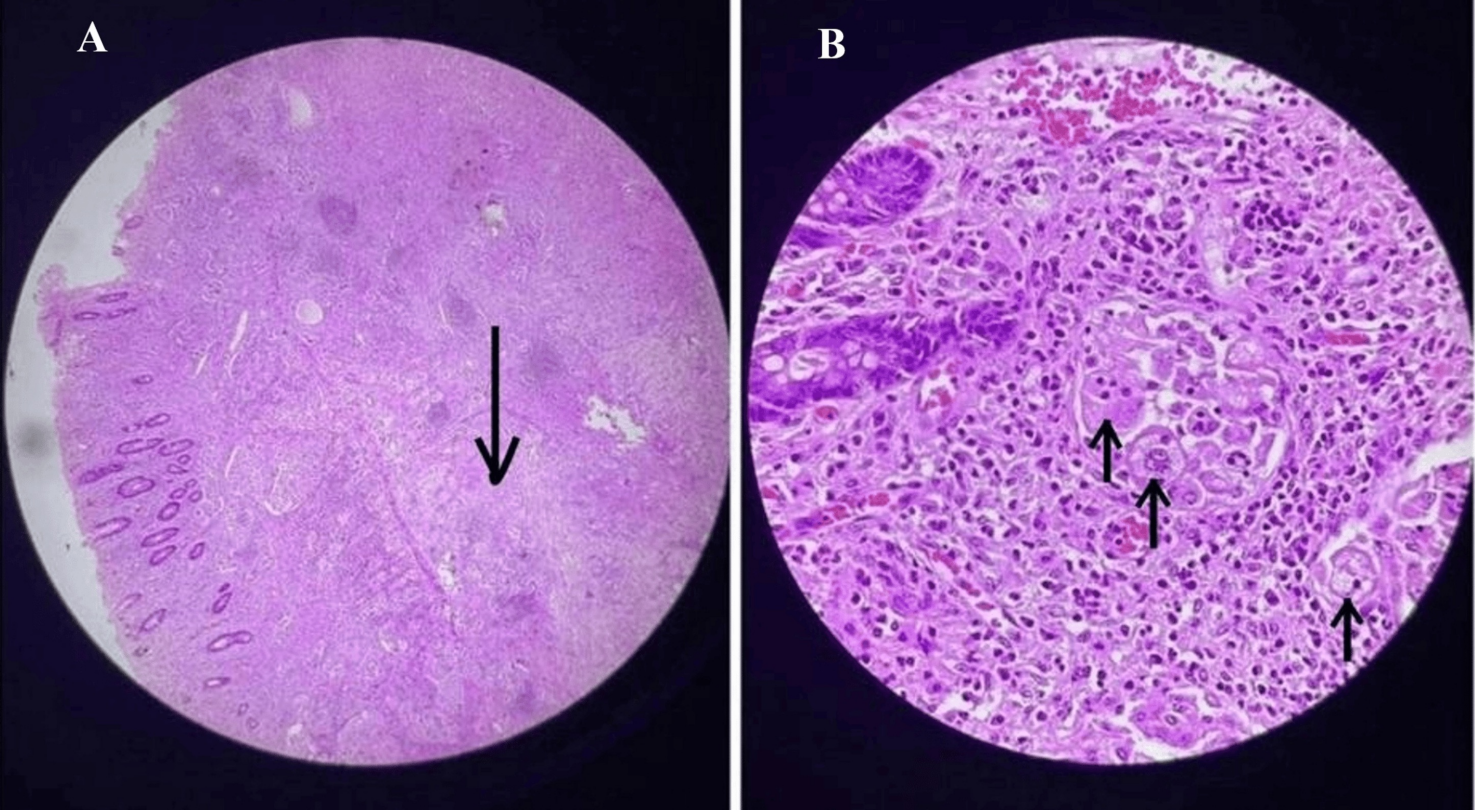
Microscopy image showing histiocytic disease involving the entire colon (transmurally), mesentery and regional lymph nodes. (A) Colon (H&E stained, 100 x magnification): submucosa is infiltrated by histiocytes (arrow); (B) (H&E stained: 400x magnification): histiocytes showing emperipolesis

Total abdominal colectomy with ileoanal anastomosis using a circular stapler and a gastro-jejunostomy. The specimen was sent for histopathological examination. The patient tolerated the procedure well. The gross histopathological examination showed irregular, yellowish, homogenous wall thickening extending from the mucosa to the serosa with no evidence of any haemorrhage or necrosis. Focal areas of infarction were noted. Microscopy revealed histiocytic disease involving the entire colon (transmurally), mesentery, and regional lymph nodes. The lesion was composed of sheets of large histiocytes with abundant foamy cytoplasm and vesicular nuclei. Numerous histiocytes also showed emperipolesis (Figure 4).

Abundant lymphoplasmacytic infiltration with extensive areas of fibrosis was seen with no mitotic activity. Immunohistochemistry revealed that histiocytes were positive for S100 and CD68 but negative for c-kit. This confirmed the diagnosis of extranodal colonic RDD. The patient suffered no postoperative complications, and he was subsequently discharged. He continues to do well four years after the operation with no evidence of disease.

## Discussion

Rosai and Dorfman first established sinus histiocytosis with massive lymphadenopathy (SHML or RDD) as a separate clinicopathological entity in 1969 [1]. It is primarily a disease of childhood and young adults and is characterised by insidious, slow progressive, painless lymphadenopathy, which is self-limiting [2]. Multisystem involvement is generally associated with poor prognosis. The Histiocyte Society's writing group recently updated the classification of RDD [4]. RDD has been divided into the following subtypes: extranodal, neoplasia-associated, familial, classical, and immunological disease-associated. Extranodal RDD is seen in less than 25% of cases. Every organ system can be affected by RDD, but the most commonly affected organs in the extranodal subtype include skin and soft tissues, the upper respiratory tract, and the skeletal system, followed by the genitourinary and lower respiratory tracts and the oral cavity. The GI tract is the least commonly involved site [3].

Table 1 shows the summary of 12 reported cases of extranodal RDD involving the GI system reported in English-language journals. These comprised four male and eight female patients. Two were paediatric, eight were middle-aged adults, and only two were elderly patients (over 60 years). Six cases were symptomatic with abdominal pain, abdominal mass, hematochezia, or chronic constipation. Four cases had extranodal involvement. The distal GI tract was most commonly affected. Out of 12 cases, eight underwent endoscopic evaluation, and three were diagnosed as RDD. Endoscopic procedures carried out were polypectomy, snare biopsy, and colonoscopy [5-7]. Surgical procedures carried out were right hemicolectomy, left hemicolectomy, segmental bowel resection, low anterior resection, appendecectomy, local resection, and total colectomy with gastrojejunostomy. A 4 cm transrectal mass was locally excised in one case. One case was diagnosed at autopsy. The GI sites involved were: isolated small bowel (n=1), isolated proximal colon (n=3), isolated distal colon (n=5), entire colon (n=2), colon with duodenum (n=1) [8-11].

**Table 1:** Summary of reported cases of extranodal RDD involving the GI system reported in English language journals. RDD: Rosai-Dorfman Disease; AWD: alive with disease; DOD: died of disease; R: remission; NA: not available.

Sr.No	References	Age (years)	Sex	GI Site Involved	Other Extranodal Sites	Diagnosis Done on	Treatment Given	Outcome
1	Anders et al., 2003 [1]	51	Female	Sigmoid colon	Lymph nodes	Post-surgery specimen	Low anterior colon resection	AWD, 15 months
2	Lauwers et al., 2000 [2]	56	Female	Rectum	None	Colonoscopy	NA	NA
3	Lauwers et al., 2000 [2]	13	Male	Jejunum	Bone	Autopsy	NA	DOD
4	Lauwers et al., 2000 [2]	7	Female	Appendix	Eye (cornea)	Post-surgery specimen	Appendectomy	AWD, 11 years
5	Lauwers et al., 2000 [2]	4	Male	Appendix	None	Colonoscopy	Segmental bowel resection	AWD, 12 years
6	Delacrétaz et al., 1991 [3]	60	Female	Rectum	sigmoid mesocolon, abdominal wall	Post-surgery specimen	Transrectal excision of the mass	AWD, 4.5 years
7	Long et al., 2007 [5]	79	Male	Ascending & descending colon	LNs, multiple relapse	Colonoscopy	Left Hemicolectomy	DOD
8	Nathwani et al., 2008 [6]	50	Female	Rectum	None	Snare biopsy	NA	NA
9	Ide et al., 2010 [7]	62	Female	Descending colon	None	Colonoscopic polypectomy	NA	NA
10	Osborne et al., 1981 [8]	53	Female	Colon	Peritoneum	Post-surgery specimen	Right hemicolectomy	AWD, 17 months
11	Shukla et al., 2003 [9]	55	Female	Caecum	None	Post-surgery specimen	Local resection	NA
12	Current case	47	Male	Whole Colon with duodenum	Lymph nodes	Post-surgery specimen	Total colectomy with ileo-anal anastomosis with Gastrojejunostomy	R, 4 years

The diagnosis of RDD is faced with great difficulty because its morphological and immunophenotypic features are somewhat similar to reactive and neoplastic diseases. The differentials include Hodgkin’s lymphoma, metastasis from various neoplasms and melanomas, malignant histiocytic neoplasm, and other inflammatory diseases. Definite diagnosis of extranodal RDD can be made by appreciating the presence of inflammatory cells and histiocytes that show emperipolesis and immunohistochemistry positive for S-100 and CD68.

The cause of RDD has been hypothesised as immune dysfunction, but it still remains unclear. The RDD histiocytes exhibit an extensive array of markers, including the S-100 protein, pan-macrophage antigens, and phagocytosis-related antigens. Nguyen et al. demonstrated that histiocytes in RDD express CD163, an acute phase-regulated transmembrane protein and hemoglobin scavenger receptor present on tissue macrophages and monocytes [12]. This finding supports the assumption that the dominant cell in RDD is activated by macrophages and is distinct from Langerhans’ cells and dendritic cells. But the definite mechanism of RDD is yet to be proven. In addition, parvovirus B19 and human herpes virus-6 have been hypothesized as contributing factors to the pathophysiology of RDD [13-15].

The closest differentials of RDD are GI xanthoma and Langerhans’ cell histiocytosis. Xanthoma presents histologically as foamy histiocytes that express CD68. As per a study by Nakasono et al. of 25 cases of rectosigmoid xanthomata [15], the histiocytosis was restricted to the lamina propria, unlike in RDD, and did not extend into the submucosa. Most of the histiocytes contained neither cell debris nor pigments in cytoplasm, and mild lymphoplasmacytic infiltration was present, which is in contrast to RDD. Langerhans’ cell histiocytosis can be differentiated from RDD by testing immunoreactivity to S-100 and CD1a; while Langerhans’ cell histiocytosis is reactive to both S-100 and CD41a, RDD is only reactive to S-100.

Treatment for RDD is not well-defined. In most patients, the disease is self-limiting. In asymptomatic patients or in patients who don’t present with involvement or compression of any major organs, no active intervention is done, and patients are asked to follow up for observation. Surgical resection is helpful in symptomatic cases with involvement of major organs.

Studies by Antonius et al. [16] and Ocheni et al. [17] suggest the use of oral corticosteroids for RDD, which has not been proven. In our case, surgical resection of the affected organ, i.e., the colon, provided symptomatic relief with no evidence of any further progression or relapse of the disease on follow-up of four years.

## Conclusions

Extranodal RDD, which affects the entire colon, is an extremely rare clinical entity that presents substantial diagnostic limitations due to its inconsistent presentation and histological overlap with other inflammatory and neoplastic disorders. Histopathology and immunohistochemistry must be used for early detection in order to prevent misdiagnosis and guide subsequent necessary surgical intervention. The present case emphasizes the need to include RDD in the differential diagnosis of diffuse colonic lesions and contributes to the limited literature on this unusual manifestation.
